# *Notes from the Field*: HeatRisk Forecasts and Emergency Department Visits for Heat-Related Illness — New York, May–September 2024

**DOI:** 10.15585/mmwr.mm7418a2

**Published:** 2025-05-22

**Authors:** Neil A. Muscatiello, Wanhsiang Hsu, Heather Aydin-Ghormoz, Charlene Weng, Vajeera Dorabawila, Kathleen F. Bush, Ambarish Vaidyanathan

**Affiliations:** ^1^New York State Department of Health; ^2^Division of Environmental Health Science and Practice, National Center for Environmental Health, CDC.

**Summary**

**What is already known about this topic?**

Exposure to heat is associated with adverse health outcomes including heat-related illness (HRI).

**What is added by this report?**

In this ecological analysis, the HeatRisk index, a color and number index developed by the National Weather Service in partnership with CDC to communicate heat-related health risk, was associated with higher rates of HRI emergency department visits in New York (outside of New York City), during May–September 2024.

**What are the implications for public health practice?**

The association between HeatRisk forecasts indicating higher levels of risk and higher HRI rates in New York (outside of New York City) supports the use of HeatRisk as a tool to increase awareness about heat exposure.

## Introduction

From 1901 to 2022, average temperatures in New York increased by approximately 2.6°F (1.4°C) (*1*). In New York, heat preparedness measures have included assessing associations between heat and health outcomes (*2*), calibrating heat advisory thresholds based on health effects, and building partnerships to bolster heat mitigation and adaptation. During spring 2024, the National Weather Service, in partnership with CDC, released HeatRisk for the continental United States. The HeatRisk index* provides a health-based heat forecast up to 7 days in advance of hot weather; the 5-level color scale (range = 0 [green]: little to no risk to 4 [magenta]: extreme risk for heat-related impact) accounts for the unique heat-related health risks in different places and times of year, and the heat duration, including both daytime and nighttime temperatures (*3*,*4*).

## Investigation and Outcomes

During May–September 2024, the New York State Department of Health (NYSDOH) evaluated HeatRisk 24-hour forecasts and associated heat-related illness (HRI). Daily county-level HeatRisk forecasts were downloaded^†^ for the 57 New York counties (outside of New York City [NYC]). NYSDOH Electronic Syndromic Surveillance System^§^ HRI emergency department (ED) visits were defined as those with *International Classification of Diseases, Tenth Revision* codes T67 (effects of heat and light), X30 (exposure to excessive natural heat–hyperthermia), L55 (sunburn), X32 (exposure to sunlight), or relevant chief complaints (e.g., sunstroke, heatstroke, heat exhaustion, or sunburn). HRI ED visits were aggregated to county-day^¶^ counts and linked with the county-day HeatRisk forecasts and county population.** For each region,^††^ and for New York (outside of NYC), HRI rates were calculated by summing the total number of HRI ED visits and dividing by the cumulative region or county population for each HeatRisk level (i.e., little to no risk, minor risk, moderate risk, major risk, or extreme risk for HRI). This project represents public health practice by NYSDOH, and institutional review board review was not required. This activity was also reviewed by CDC, deemed not research, and was conducted consistent with applicable federal law and CDC policy.^§§^

Across all regions, at least 79% of county-days had HeatRisk forecasts in the little to no risk or minor risk levels, and none had more than 0.3% of county-days in the extreme risk level (Figure). In six of the seven regions and in New York (outside of NYC), HeatRisk forecasts indicating a higher level of risk were associated with higher HRI rates (Figure). In the Capital District region, no HRI ED visits occurred on the few county-days in the extreme risk level, and in the Hudson Valley, Long Island, and Northeast regions, no county-days had an extreme risk for HRI, although each region’s HRI rate increased with increasing HeatRisk forecast through the major risk levels.

**FIGURE:**
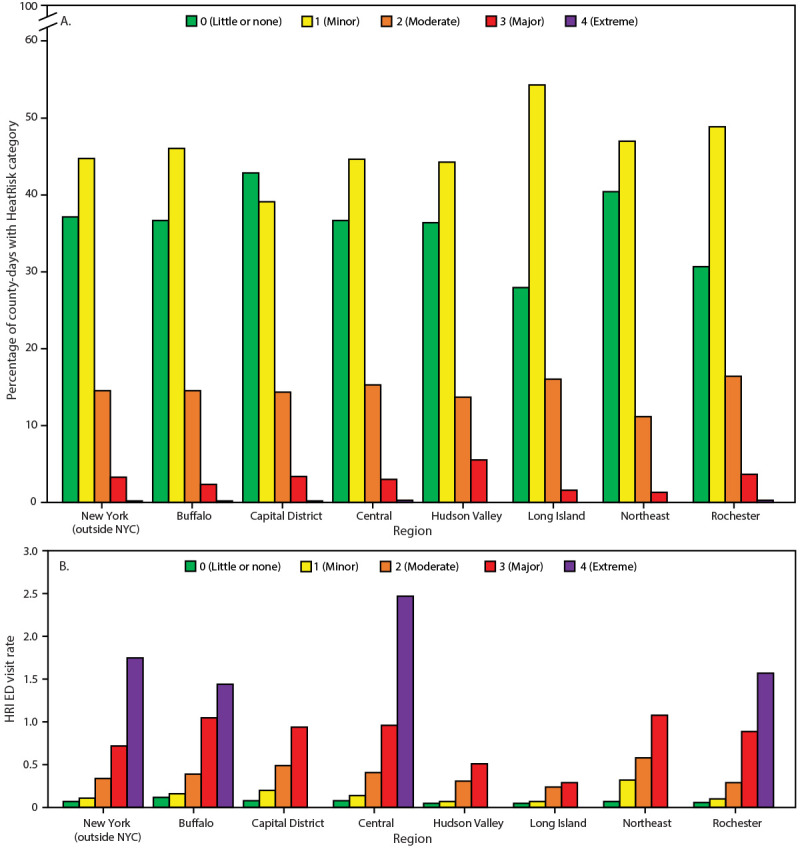
Percentage of county-days* with heat-related risks (A) and emergency department visit rates^†^ for heat-related illness (B), by HeatRisk level^§^ and region^¶^ — New York, May–September 2024 **Abbreviations:** ED = emergency department; HRI = heat-related illness; NYC = New York City. * Number of county-days in HeatRisk level for region divided by total county-days in region. A county-day represents each combination of county and day for the study period (i.e., for the 57 counties, each day during May–September represents 57 county-days). ^†^ HRI ED visits per 100,000 population. ^§^ National Weather Service | HeatRisk (Accessed May 16, 2025). ^¶^ Total number of counties = 57; by region: Buffalo (eight), Capital District (13), Central (13), Hudson Valley (seven), Long Island (two), Northeast (five), and Rochester (nine).

## Preliminary Conclusions and Actions

The findings suggest that HeatRisk forecasts can be used to increase awareness about health risks from heat exposure, provide a resource for state and local agencies to implement response actions, and empower the public and public health professionals to take steps to minimize exposure. The accessibility of HeatRisk might be improved through incorporation into mobile device weather apps. Further study is warranted to explore possible uses for HeatRisk in preventing heat-related harm (e.g., as an early-warning tool to prevent heat-related adverse health outcomes).

## Footnotes

* How to use the HeatRisk Tool and Air Quality Index | Heat Health | CDC

† National Environmental Public Health Tracking Network Data Explorer (Accessed May 16, 2025).

§
Electronic Syndromic Surveillance System (ESSS)

¶ A county-day represents each combination of county and day for the study period (i.e., for the 57 counties, each day during May–September represents 57 county-days).

**
American Community Survey 5-Year Data (2009–2023)

†† Regions were adapted from county groupings used by the Electronic Syndromic Surveillance System. *Buffalo *(eight counties): Allegany, Cattaraugus, Chautauqua, Erie, Genesee, Niagara, Orleans, and Wyoming; *Capital District* (13 counties): Albany, Columbia, Delaware, Fulton, Greene, Montgomery, Otsego, Rensselaer, Saratoga, Schenectady, Schoharie, Warren, and Washington; *Central* (13 counties): Broome, Cayuga, Chenango, Cortland, Herkimer, Jefferson, Lewis, Madison, Oneida, Onondaga, Oswego, Tioga, and Tompkins; *Hudson Valley* (seven counties): Dutchess, Orange, Putnam, Rockland, Sullivan, Ulster, and Westchester; *Long Island* (two counties): Nassau and Suffolk; *Northeast* (five counties): Clinton, Essex, Franklin, Hamilton, and St. Lawrence; and *Rochester* (nine counties): Chemung, Livingston, Monroe, Ontario, Schuyler, Seneca, Steuben, Wayne, and Yates.

§§ 45 C.F.R. part 46.102(l)(2), 21 C.F.R. part 56; 42 U.S.C. Sect. 241(d); 5 U.S.C. Sect. 552a; 44 U.S.C. Sect. 3501 et seq.
